# Health outcomes and unmet needs in patients with long-standing rheumatoid arthritis attending tertiary care in Greece: a cohort study

**DOI:** 10.1186/s12955-019-1127-8

**Published:** 2019-04-29

**Authors:** Dimitrios T. Boumpas, Prodromos Sidiropoulos, Loukas Settas, Piotr Szczypa, Vassilis Tsekouras, Ana C. Hernandez Daly

**Affiliations:** 1 School of Medicine, National and Kapodistrian University of Athens, Athens, Greece; 2 School of Medicine, University of Crete, Heraklion, Crete Greece; 3 Aristotle University of Thessaloniki, Thessaloniki, Greece; 4 Pfizer Ltd, Walton Oaks, UK; 5 Pfizer Hellas, Athens, Greece; 6 ATTIKON University Hospital, 1 Rimini Str, Chaidari, 124 62 Athens, Greece

**Keywords:** Rheumatoid arthritis, Quality of life, Patient-reported outcomes, Disease-modifying antirheumatic drug, Health assessment questionnaire, Euro quality of Life-5 dimensions questionnaire, Unmet need

## Abstract

**Background:**

No previous studies have characterized a patient’s experience of rheumatoid arthritis (RA) management in Greece and unmet needs may exist despite a broad range of available treatments. Therefore, we assessed quality of life (QoL), functional ability, and healthcare resource utilization in patients with established RA and receiving treatment in a tertiary care setting in Greece.

**Methods:**

This was a prospective, observational cohort of patients aged ≥18 years, receiving any type of treatment for RA, and followed for 12 months at 7 rheumatology referral centers across mainland Greece (NCT01001182). Patient data were collected at the initial visit and 3, 6, and 9 months. QoL was evaluated using the Euro Quality of Life-5 dimensions questionnaire (EQ-5D) and functional ability was evaluated using the Health Assessment Questionnaire (HAQ).

**Results:**

A total of 210 patients with RA were enrolled (76.7% women, mean ± standard deviation [SD] age: 59.1 ± 12.6 years, median [interquartile range] disease duration: 11.9 [5.0–16.0] years). Baseline mean ± SD EQ-5D and HAQ scores were 0.57 ± 0.32 and 0.75 ± 0.63, respectively, and remained largely unchanged throughout the study. Post-hoc comparison showed that patients receiving non-biologic disease-modifying antirheumatic drugs (non-bDMARDs) had significantly higher EQ-5D and lower HAQ-DI scores compared with those receiving biologic DMARDs. A majority of patients reported having difficulty doing housework or other duties (61.4 and 61.9%, respectively), and 55.2% reported needing external support for these tasks. Positive correlation was observed between QoL and functional ability. Hospitalization at least once during the study occurred in 9.5% of the patients, and 12.5% of these cases were due to exacerbation of RA. At baseline, 52.4% of the patients were retired, with 38.5% of retirees having retired early due to RA. Among the patients who were retired at baseline, the mean ± SD period from actual retirement to expected retirement age was 12.1 ± 8.1 years.

**Conclusion:**

QoL and functional ability were positively correlated in patients with long-standing RA, with a large proportion showing impairments in both. Timely, target-oriented treatment initiated as soon as possible after diagnosis may help to improve patient-reported outcomes and limit the burden of RA.

**Trial registration:**

ClinicalTrials.gov NCT01001182. Registered 23 October 2009.

**Electronic supplementary material:**

The online version of this article (10.1186/s12955-019-1127-8) contains supplementary material, which is available to authorized users.

## Background

Rheumatoid arthritis (RA) is an autoimmune disease that causes inflammation and progressive destruction of the joints, leading to functional disability and potentially to partial immobility and/or severe pain [1]. The pain and fatigue associated with RA, in addition to the loss of function caused by joint damage, negatively impact a patient’s quality of life (QoL). Patients with RA can experience difficulties in performing simple everyday tasks [2–4], which can in turn affect their ability to care for themselves to such an extent that they require external support. The loss of physical function in patients with RA can also cause work-related challenges, such as decreased productivity or early retirement due to an inability to work [3–5]. Furthermore, patients with RA have an increased risk of developing comorbidities [6], including cardiovascular diseases, respiratory infections, and malignancies. These comorbidities occur in patients with RA partly as a side effect of the medications commonly used to treat the disease [7] and partly as a consequence of the ongoing inflammatory activity [8]. Overall, the burden of the disease has a severe impact on the physical and emotional well-being of individual patients, as well as on society as a whole [9, 10].

Currently available therapies for RA aim to induce remission or low disease activity in order to reduce disability, limit the negative impact on QoL [11, 12], and control the overall utilization of healthcare resources associated with management of the disease. The use of conventional synthetic disease-modifying antirheumatic drugs (csDMARDs) is recommended as first-line therapy, followed by the use of combination therapy with csDMARDs and either biologic DMARDs (bDMARDs) or targeted synthetic DMARDs (tsDMARDs) in patients who fail to respond to csDMARDs [13, 14]. However, factors able to predict a patient’s response to a specific treatment have not yet been identified and therefore current therapies may not always perfectly match an individual’s needs, with treatment adjustment or switching often required in non-responders.

If appropriate control of RA is not achieved in a timely manner then patients are unlikely to attain optimal disease status [14]. A recent study has shown that a shorter time to remission is associated with a longer period of sustained remission [15]. If the treatment goal for patients with RA is set as early remission or low disease activity then they should experience less joint damage and less disease progression [16], which will also be maintained for a longer period of time. The findings from these studies suggest that treatment initiation at the appropriate time during the disease course is crucial for achieving optimal health outcomes.

To the authors’ knowledge, there have been no previous studies of patients with RA in Greece that have assessed the impact of available treatments and treatment strategies on QoL, disease-related parameters, and the ability of patients to care for themselves and work. Therefore, we conducted an observational, non-interventional study to evaluate these three variables in patients with RA attending Greek tertiary referral centers and then performed a subsequent post-hoc comparison of data from patients receiving treatment with bDMARDs with data from patients receiving non-bDMARDs.

## Methods

### Study design and patients

This study was conducted in Greece between June 2010 and April 2012, and was a prospective, observational epidemiological cohort of patients with RA at any disease stage. Patients aged ≥18 years of age and receiving any treatment for RA were eligible for inclusion and were enrolled from 7 rheumatology clinics in academic hospitals and regional referral centers throughout mainland Greece. Patients were also required to have a confirmed diagnosis of RA by the investigating physician, which was made according to the usual practice at each of the study sites, in order to be included. Patients were excluded from the study if they were enrolled in any simultaneous, interventional clinical trials. Patients were sequentially enrolled in the order that they visited the clinic until the predefined number of patients was reached at each site. A total of 210 patients was initially planned to be recruited into the study, which was designed to be as inclusive as possible in order to evaluate as many patients at the participating study centers as possible; the number of patients to be included in the study was not derived from formal power calculations. Patient data were collected at an initial evaluation (baseline) and at 3 follow-up visits every 3 months, and therefore the observational period for each patient was 9 months. To capture a 12-month period, retrospective data related to study variables within 3 months prior to enrollment were collected during the baseline visit.

The study was conducted in accordance with the ethical principles of the Declaration of Helsinki and according to generally accepted research practices such as those described in the Good Pharmacoepidemiology Practices issued by the International Society for Pharmacoepidemiology, the guidelines of the International Society for Pharmacoeconomics and Outcomes Research, and the guidelines of the Pharmaceutical Research and Manufacturers Association. All patients gave written informed consent, which was compliant with the International Conference on Harmonisation guideline for Good Clinical Practice and which was reviewed and approved by an independent ethics committee or institutional review board, and could request to withdraw from the study at any time, or could be withdrawn at any time at the discretion of the investigator or sponsor for safety, behavioral, or administrative reasons. This study (B1801120) was approved by the Greek Regulatory Authorities (approval numbers 85,282 [14 December 2009] and 36,404 [9 June 2010]) and is registered with ClinicalTrials.gov (NCT01001182).

### Variables

Patient demographics and medical histories were recorded using an Investigator’s Questionnaire, and included date of birth, gender, height, weight, smoking history, alcohol consumption, level of education, type of health insurance, and medical and RA history. Healthcare utilization (including medication received for RA during the study), number and location of visits to physicians, specialty of treating physicians, number and type of diagnostic tests conducted (which could be conducted at any time during the study period, for example as an outpatient or upon hospitalization), and the number of hospitalizations and surgical procedures were also recorded.

A Patient’s Questionnaire was used to obtain data on QoL and functional ability. QoL was evaluated using a validated Greek translation of the Euro Quality of Life-5 dimensions questionnaire (EQ-5D) [17, 18]. Utilities were derived from internationally developed and validated tables [2]. The functional ability of patients was evaluated using a validated Greek translation of the Health Assessment Questionnaire (HAQ) [19]. HAQ score banding was specified according to international practice [20, 21]. Indirect healthcare resource utilization included reasons for premature retirement (where applicable), problems at/with work due to RA, absenteeism from work, problems with everyday tasks due to RA, and the provision of unpaid care. Among the patients who were employed workers, absenteeism and presenteeism was evaluated and included the number of days absent from work and the number of days with reduced working hours. The EQ-5D, HAQ, and resource utilization questionnaires were completed at each 3-month visit until data for 1 year had been collected. A degree of bias relating to the fact that patients were recruited from referral centers where those with more severe RA phenotypes are followed was anticipated. The possibility of reviewer bias from respondents was avoided using standardized questionnaires validated in the Greek population. The questionnaires were completed during the patients’ visits to the referral centers and not by mail.

### Statistical analysis

One-sample t-tests were applied to evaluate the statistical significance of changes in EQ-5D and HAQ scores between baseline and Month 9 within each patient subgroup. Mixed-effects models were used to account for confounding factors that could highlight any differences in EQ-5D or HAQ-DI score between patients who received a bDMARD and those who did not. Time and the use of a bDMARD were fixed effects, and age, gender, body mass index, smoking status, alcohol consumption, and time from diagnosis were assessed as covariates. The restricted-maximum-likelihood method was used to estimate the fixed effects of the model and the significance of covariates. A mixed-effects model with only the statistically significant covariates and interaction terms was implemented respectively to improve model fit. Statistical analyses were performed using SAS v9.2 software. The Kruskal–Wallis test was used to compare differences in Health Assessment Questionnaire-Disability Index (HAQ-DI) score distribution between patients with different occupational status at each study visit. Missing data were not imputed except in the event that questionnaire data (EQ-5D or HAQ) were missing at a particular visit, in which case information was extracted from the previous questionnaire using the last-observation-carried-forward method. In order to be included in the analysis, patients had to complete at least 3 of the 4 questionnaires, including the baseline questionnaire. Results for quantitative variables were expressed as mean ± standard deviation (SD) unless otherwise stated.

## Results

### Baseline characteristics

The number of patients attending the baseline, 3, 6, and 9 months study visits was 210, 184, 195, and 198, respectively (reasons for discontinuation were not recorded). The baseline demographic characteristics of the overall cohort, as well as patients grouped according to whether they had received prior bDMARD, are shown in Table 1. In the overall cohort, the median time from first diagnosis of RA was 11.9 years (interquartile range: 5.0–16.0), the mean ± SD total EQ-5D score was 0.57 ± 0.32, and the mean ± SD total HAQ score was 0.75 ± 0.63. Most diagnoses of RA were made by a rheumatologist (79.8%) or an internist (14.4%). The occupational status of the patients is shown in Table 2. Among the patients who were retired at baseline, for whatever reason, the mean ± SD period of time from actual retirement to the expected retirement age was 12.1 ± 8.1 years.

**Table 1:** Patient demographics and social characteristics

Characteristics	Overall	Non-bDMARDs *n* = 87	bDMARDs *n* = 123	*p*-value
Sex, *n (%)*	*N* = 210			
Male	49 (23.3)	23 (26.4)	26 (21.1)	0.372
Female	161 (76.7)	64 (73.6)	97 (78.9)	
Age, (years)	*N* = 210			
Mean ± SD	59.1 ± 12.6	60.38 ± 13.05	57.32 ± 12.21	0.083
IQR	51.8–67.8	55.0–68.0	49.0–67.0	
Weight, (kg)	*N* = 187			
Mean ± SD	76.0 ± 14.8	NA	NA	
IQR	65.0–85.0			
BMI, (k/m^2^)	*N* = 174			
Mean ± SD	28.1 ± 4.6	28.37 ± 4.39	27.92 ± 4.82	0.534
IQR	24.9–30.5	25.5–31.2	24.7–30.3	
Education, *n* (%)	*N* = 210			
No education	2 (1.0)	NA	NA	
Primary	113 (53.8)			
Secondary	63 (30.0)			
Higher	32 (15.2)			
Smoker, *n* (%)	*N* = 210			
Never smoked	147 (70.0)	61 (70.1)	86 (69.9)	0.848
Ex-smoker	32 (15.2)	15 (17.2)	17 (13.8)	
Time since stopped (years), Mean ± SD (*n*)	9.9 ± 10.5 (30)			
Packs/year, Mean ± SD (*n*)	30.1 ± 40.8 (25)			
Smoker	31 (15)	11 (12.6)	20 (16.3)	
Packs/year, Mean ± SD (*n*)	18.7 ± 14.1 (28)			
Patient diagnosed for the first time	*N* = 210			
No, *n* (%)	208 (99.0)	85 (97.7)	123 (100)	0.092
Time from first diagnosis (years), median (IQR)	11.9 (5.0–16.0)	10.0	11.0	
Yes, *n* (%)	2 (1.0)	2 (2.3)	0 (0)	0.469
Duration of symptoms (months), median (IQR)	5.5 (4.0–7.0)			

*bDMARD* biologic disease-modifying antirheumatic drug, *BMI* body mass index, *IQR* interquartile range, *SD* standard deviation

**Table 2:** Patient occupational status

Occupational status, *n* (%)	Baseline *N* = 210	3 months *N* = 183	6 months *N* = 195	9 months *N* = 198
Employed	38 (18.1)	33 (18.0)	35 (17.9)	34 (17.2)
Unemployed	9 (4.3)	6 (3.3)	6 (3.1)	5 (2.5)
Housekeeping	50 (23.8)	40 (21.9)	48 (24.6)	48 (24.2)
Retired	110 (52.4)	102 (55.7)	104 (53.3)	109 (55.1)
At normal age	47 (43.1)^a^	42 (41.6)^a^	45 (43.7)^a^	46 (42.6)^a^
Earlier, due to RA	42 (38.5)^a^	42 (41.6)^a^	42 (40.8)^a^	44 (40.7)^a^
Earlier, due to RA & other reasons	2 (1.8)^a^	2 (2.0)^a^	2 (1.9)^a^	2 (1.9)^a^
Earlier, due to other reasons	18 (16.5)^a^	15 (14.9)^a^	14 (13.6)^a^	16 (14.8)^a^
Not applicable	1 (0.9)^a^	1 (1.0)^a^	1 (1.0)^a^	1 (0.9)^a^
Not specified	3 (1.4)	2 (1.1)	2 (1.0)	2 (1.0)

*RA* rheumatoid arthritis

^a^Percentage of retired patients

More than half of the patients had > 1 comorbidity, with the most frequent being hypertension (46.7%), followed by osteoporosis, hypothyroidism, dyslipidemia, diabetes, and depression (prevalence: 11.4–16.7% for each). All other comorbidities occurred with a frequency ≤ 6.2%.

The mean ± SD duration of prior treatment was longest in those receiving csDMARDs (69.3 ± 68.4 months), followed by analgesics (48.0 ± 72.4 months), NSAIDs (35.1 ± 37.6 months), and bDMARDs (24.1 ± 24.8 months). The bDMARD with the longest mean ± SD duration of treatment was infliximab (40.9 ± 31.4 months), followed by etanercept (22.2 ± 20.4 months), abatacept (21.8 ± 10.1 months), adalimumab (20.3 ± 20.5 months), anakinra (9.7 ± 12.1 months), tocilizumab (7.0 ± 8.5 months), rituximab (5.1 ± 4.1 months), and golimumab (5.0 ± 1.4 months). The most frequent reasons for patient discontinuation of a prior bDMARD was lack of efficacy (61.8%) and incident adverse events (23.6%). Age was the only baseline covariate that was significantly different between patients in the bDMARD and non-bDMARD subgroups (*p* = 0.015 at the Month 3 visit and *p* = 0.025 at the Month 6 visit).

### Impact of established RA on QoL

**Fig. 1:**
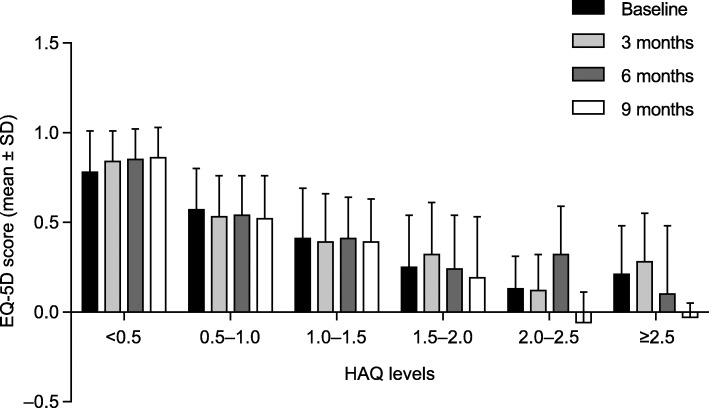
Presentation of EQ-5D questionnaire results according to HAQ category. Abbreviations: *EQ-5D* Euro Quality of Life-5 dimensions questionnaire, *HAQ* Health Assessment Questionnaire, *SD* standard deviation

The mixed-effect model of EQ-5D accounted for the effect over time for different subgroups. The adjusted least-squares mean ± standard error total EQ-5D score at 9 months was 0.49 ± 0.03 for patients in the bDMARD subgroup and 0.65 ± 0.04 for patients in the non-bDMARD subgroup (Table 3). Throughout the study period, the dimensions with the highest negative rates were anxiety/depression and pain/discomfort. In the mixed-effect model fitted across time points for EQ-5D, there was a significant difference in total EQ-5D scores between the bDMARD and non-bDMARD subgroups (estimate: 0.107, 95% confidence interval [CI]: 0.066, 0.148; *p* < 0.0001), while patients who were non-smokers or ex-smokers (estimate: 0.033, 95% CI: 0.004, 0.060; *p* = 0.021) had a higher EQ-5D score compared with smokers, and women (estimate: − 0.195, 95% CI: − 0.243, − 0.146; *p* < 0.0001) had lower EQ-5D scores compared with men (Additional file 1: Table S1). A positive relationship between QoL (as assessed by mean EQ-5D scores) and functional ability (as assessed by mean HAQ scores) was observed (Fig. 1).

**Table 3:** EQ-5D scores at each study visit according to treatment received (Level 1 = no problem; Level 2 = medium; Level 3 = severe)

EQ-5D	Baseline		3 months		6 months		9 months	
	Non-bDMARDs	bDMARDs	Non-bDMARDs	bDMARDs	Non-bDMARDs	bDMARDs	Non-bDMARDs	bDMARDs
Mobility, *n* (%)	*N* = 86	*N* = 123	*N* = 68	*N* = 115	*N* = 73	*N* = 121	*N* = 78	*N* = 119
Level 1	43 (50.0)	50 (40.7)	40 (58.8)	45 (39.1)	49 (67.1)	48 (39.7)	47 (60.3)	54 (45.4)
Level 2	43 (50.0)	72 (58.5)	28 (41.2)	70 (60.9)	24 (32.9)	73 (60.3)	31 (39.7)	63 (52.9)
Level 3	0 (0.0)	1 (0.8)	0 (0.0)	0 (0.0)	0 (0.0)	0 (0.0)	0 (0.0)	2 (1.7)
Self-care, *n* (%)	*N* = 86	*N* = 123	*N* = 68	*N* = 115	*N* = 73	*N* = 121	*N* = 78	*N* = 119
Level 1	60 (69.8)	77 (62.6)	45 (66.2)	68 (59.1)	45 (61.6)	63 (52.1)	46 (59.0)	61 (51.3)
Level 2	26 (30.2)	46 (37.4)	23 (33.8)	47 (40.9)	28 (38.4)	58 (47.9)	32 (41.0)	56 (47.1)
Level 3	0 (0.0)	0 (0.0)	0 (0.0)	0 (0.0)	0 (0.0)	0 (0.0)	0 (0.0)	2 (1.7)
Usual activities, *n* (%)	*N* = 85	*N* = 123	*N* = 68	*N* = 115	*N* = 73	*N* = 121	*N* = 78	*N* = 119
Level 1	33 (38.8)	39 (31.7)	34 (50.0)	41 (35.7)	34 (46.6)	37 (30.6)	35 (44.9)	32 (26.9)
Level 2	50 (58.8)	84 (68.3)	34 (50.0)	73 (63.5)	39 (53.4)	82 (67.8)	43 (55.1)	85 (71.4)
Level 3	2 (2.4)	0 (0.0)	0 (0.0)	1 (0.9)	0 (0.0)	2 (1.7)	0 (0.0)	2 (1.7)
Pain/discomfort, *n* (%)	*N* = 86	*N* = 123	*N* = 68	*N* = 115	*N* = 73	*N* = 121	*N* = 78	*N* = 119
Level 1	28 (32.6)	33 (26.8)	28 (41.2)	25 (21.7)	33 (45.2)	25 (20.7)	38 (48.7)	25 (21.0)
Level 2	56 (65.1)	80 (65.0)	38 (55.9)	84 (73.0)	39 (53.4)	88 (72.7)	36 (46.2)	82 (68.9)
Level 3	2 (2.3)	10 (8.1)	2 (2.9)	6 (5.2)	1 (1.4)	8 (6.6)	4 (5.1)	12 (10.1)
Anxiety/depression, *n* (%)	*N* = 86	*N* = 123	*N* = 68	*N* = 115	*N* = 73	*N* = 121	*N* = 78	*N* = 119
Level 1	31 (36.0)	43 (35.0)	30 (44.1)	42 (36.5)	38 (52.1)	34 (28.1)	37 (47.4)	28 (23.5)
Level 2	36 (41.9)	50 (40.7)	30 (44.1)	47 (40.9)	30 (41.1)	60 (49.6)	31 (39.7)	60 (50.4)
Level 3	19 (22.1)	30 (24.4)	8 (11.8)	26 (22.6)	5 (6.8)	27 (22.3)	10 (12.8)	31 (26.1)
EQ VAS score, Mean ± SD	*N* = 86	*N* = 123	*N* = 68	*N* = 115	*N* = 73	*N* = 120	*N* = 78	*N* = 119
	67.0 ± 20.2	66.0 ± 20.2	71.4 ± 20.9	67.2 ± 19.3	75.9 ± 18.8	66.7 ± 18.7	75.9 ± 19.0	67.7 ± 18.8
Total EQ-5D score, Mean ± SD	*N* = 85	*N* = 123	*N* = 68	*N* = 115	*N* = 73	*N* = 121	*N* = 78	*N* = 119
	0.60 ± 0.32	0.54 ± 0.32	0.69 ± 0.31	0.56 ± 0.30	0.73 ± 0.26	0.54 ± 0.30	0.68 ± 0.32	0.50 ± 0.34
Adjusted total EQ-5D score^a^, Least-squares mean ± SE	*N* = 85	*N* = 123	*N* = 68	*N* = 115	*N* = 73	*N* = 121	*N* = 78	*N* = 119
	0.59 ± 0.04	0.55 ± 0.03	0.66 ± 0.03	0.55 ± 0.03	0.70 ± 0.03	0.53 ± 0.03	0.65 ± 0.04	0.49 ± 0.03

*bDMARD* biologic disease-modifying antirheumatic drug, *EQ-5D* Euro Quality of Life-5 dimensions, *SD* standard deviation, *SE* standard error, *VAS* visual analog scale

^a^EQ-5D index score adjusted for baseline covariates: gender and smoking status

### Disease-related parameters during follow-up

The proportion of patients with > 1 treatment change during the study period was 8.1%, while 71.9% had no treatment change. The baseline mean ± SD total HAQ score for the overall cohort was 0.75 ± 0.63, and this remained unchanged between each study visit (0.70 ± 0.62, 0.70 ± 0.60, and 0.70 ± 0.61 at 3, 6, and 9 months, respectively; data showing the proportion of patients within different HAQ categories at each study visit are shown in Additional file 1: Table S2).

The mixed-effect model of HAQ-DI accounted for the effect over time for different subgroups. The adjusted least-squares mean ± standard error total HAQ score at 9 months was 0.78 ± 0.05 for patients in the bDMARD subgroup and 0.58 ± 0.07 for patients in the non-bDMARD subgroup (Table 4; data showing the proportion of patients within different HAQ categories at each study visit and according to the use of DMARDs are shown in Additional file 1: Table S3). In the mixed-effect model fitted across time points for HAQ-DI, patients in the non-bDMARD subgroup had a lower total adjusted HAQ-DI score compared with those in the bDMARD subgroup (estimate: − 0.186, 95% CI: − 0.272, − 0.099; *p* < 0.0001; Additional file 1: Table S4). Women (estimate: 0.359, 95% CI: 0.261, 0.458; p < 0.0001) and older patients (estimate: 0.006, 95% CI: 0.002, 0.009; *p* = 0.001) had higher HAQ-DI scores than men and younger patients, respectively. A higher body mass index was associated with a lower HAQ-DI score (estimate: − 0.009, 95% CI: − 0.016, − 0.002, *p* = 0.012). The global *p*-value for the interaction between time and body mass index was 0.001 and this interaction term was included in the final model.

**Table 4:** HAQ results over time according to use of bDMARDs

	Baseline		3 months		6 months		9 months	
	Non-bDMARDs	bDMARDs	Non-bDMARDs	bDMARDs	Non-bDMARDs	bDMARDs	Non-bDMARDs	bDMARDs
	*N* = 86	*N* = 123	*N* = 68	*N* = 115	*N* = 73	*N* = 121	*N* = 78	*N* = 119
Total HAQ score, mean ± SD	0.68 ± 0.64	0.81 ± 0.63	0.57 ± 0.62	0.75 ± 0.59	0.53 ± 0.54	0.77 ± 0.62	0.54 ± 0.53	0.78 ± 0.62
Adjusted total HAQ score^a^, Least-squares mean ± SE	0.67 ± 0.08	0.80 ± 0.07	0.58 ± 0.07	0.75 ± 0.06	0.52 ± 0.07	0.77 ± 0.05	0.58 ± 0.07	0.78 ± 0.05

*bDMARD* biologic disease-modifying antirheumatic drug, *BMI* body mass index, *HAQ* Health Assessment Questionnaire, *SD* standard deviation, *SE* standard error

^a^HAQ scores adjusted for baseline covariates: age, gender, BMI, and the interaction term of Visit^a^BMI

### Impact of established RA on working status

Among the subgroup of patients who were in employment (17.2–18.1% prevalence across the study period), 21.9–25.8% reported “working at lower tempo compared to normal” and 16.2–21.9% reported that “postponing tasks” happened “often” and “almost always” due to problems associated with having RA, although no clear overall trends were observed. Among the subgroup of patients who were employed during at least 1 time point during the study (*n* = 40), the mean duration of absence from work during the study period was 5.9 ± 16.1 days. Patients receiving a combination of csDMARD and bDMARD (*n* = 25) took a mean ± SD period of leave from work of 6.8 ± 16.8 days, whereas patients on csDMARDs alone (*n* = 11) and bDMARDs alone (*n* = 4) took 0.4 ± 1.2 days and 15.0 ± 30.0 days, respectively. Overall, the mean ± SD number of days with reduced working hours during for patients who were employed during at least 1 time point during the study (*n* = 40) was 7.3 ± 24.2 days, while the number of days in the subgroups who received a combination of csDMARD and bDMARD (*n* = 25), csDMARDs alone (*n* = 11), or bDMARDs alone (*n* = 4) were 10.4 ± 30.2, 1.7 ± 5.1, and 3.0 ± 4.8 days, respectively.

Over the study period, 61.4, 61.9, and 60.0% of the overall cohort had difficulty doing housework, external duties (e.g. shopping and paying bills), or other work (e.g. garden work and house repair), respectively. Furthermore, 55.2% of patients needed help from either their partner (57.8%), children (45.7%), a paid person (31.0%), or another individual (32.8%) to perform some of these tasks.

When disability, in terms of mean HAQ-DI scores, was compared among the different occupational subgroups, a greater proportion of employed patients had a HAQ-DI score of 0.0–1.0) than in all of the other subgroups (75.8–85.7% vs 33.3–75.0%, respectively, Table 5). A statistically significant difference in the distribution of patient occupational status among the different HAQ-DI categories was observed at baseline (*p* = 0.041) and at 6 months (*p* = 0.014).

**Table 5:** Employment status according to HAQ-DI scores during the study period

HAQ-DI score^a^, *n* (%)	Employed	Retired	Unemployed	Housekeeping	Other	*p*-value†
Baseline	*N* = 38	*N* = 110	*N* = 9	*N* = 50	*N* = 3	0.041
0.0–< 1.0	31 (81.6)	66 (60.0)	4 (44.4)	37 (74.0)	2 (66.7)	
1.0–< 2.0	6 (15.8)	39 (35.5)	3 (33.3)	12 (24.0)	0 (0.0)	
2.0–≤3.0	1 (2.6)	5 (4.5)	2 (22.2)	1 (2.0)	1 (33.3)	
3 months	*N* = 33	*N* = 102	*N* = 6	*N* = 40	*N* = 2	0.195
0.0–< 1.0	25 (75.8)	62 (60.8)	3 (50.0)	30 (75.0)	2 (100.0)	
1.0–< 2.0	7 (21.2)	35 (34.3)	2 (33.3)	9 (22.5)	0 (0.0)	
2.0–≤3.0	1 (3.0)	5 (4.9)	1 (16.7)	1 (2.5)	0 (0.0)	
6 months	*N* = 35	*N* = 104	*N* = 6	*N* = 48	*N* = 2	0.014
0.0–< 1.0	30 (85.7)	65 (62.5)	2 (33.3)	36 (75.0)	1 (50.0)	
1.0–< 2.0	5 (14.3)	35 (33.7)	3 (50.0)	11 (22.9)	0 (0.0)	
2.0–≤3.0	0 (0.0)	4 (3.8)	1 (16.7)	1 (2.1)	1 (50.0)	
9 months	*N* = 34	*N* = 109	*N* = 5	*N* = 48	*N* = 2	0.232
0.0–< 1.0	28 (82.4)	71 (65.1)	3 (60.0)	36 (75.0)	1 (50.0)	
1.0–< 2.0	6 (17.6)	33 (30.3)	2 (40.0)	10 (20.8)	0 (0.0)	
2.0–≤3.0	0 (0.0)	5 (4.6)	0 (0.0)	2 (4.2)	1 (50.0)	

*HAQ-DI* Health Assessment Questionnaire-Disability Index

^a^0 = without any difficulty; 1 = with some difficulty; 2 = with much difficulty; 3 = unable to do

†Difference in HAQ-DI scores (as categorized) between the different occupational statuses (Kruskal–Wallis Test)

### Healthcare-related resource utilization

Hospitalization at least once during the study occurred in 9.5% of the overall cohort, with exacerbation of RA being the most frequent reason (12.5% of cases). The most frequent diagnostic tests performed during the study period were blood tests (45.5%), followed by urological tests (21.5%), with a monthly median number of 0.3 and 0.2, respectively. The diagnostic tests were performed mostly in public hospitals (69.0 and 61.7%, respectively). Imaging procedures, which included plain radiography, ultrasound, magnetic resonance imaging, and computed tomography scans, accounted for 4.4% of the total number of diagnostic tests performed during the study period.

## Discussion

The present study aimed to understand a patient’s experience of current RA management and treatment outcomes in Greece by investigating the impact of the disease on QoL and functional ability rather than focusing on clinical parameters. Patient-reported outcomes are included in some composite outcome measures, such as the American College of Rheumatology responses, in order to integrate the impact that RA has on a patient’s life into the endpoints used in clinical trials and clinical practice. The results from routine clinical evaluations may not fully reflect a patient’s perception of their own health status because they do not encompass all the symptoms associated with RA, and even if patients achieve remission or low disease activity then they may still experience pain and difficulties at work and/or in their everyday life in such a way that their QoL and functional ability are severely impacted. A previous study has shown that patients involved in disease management were more satisfied and persisted with treatment [22], which highlights the importance of considering a patient’s perspective when choosing a therapy.

The present study demonstrated that, even though a broad range of treatments for RA are available in Greece, unmet needs in terms of functional ability and emotional and social well-being may still exist in patients with established RA. A failure to control RA may be attributed to various factors, such as delayed diagnosis and introduction of adequate therapies or a physician’s limited experience with such therapies, or a lack of implementation of treat-to-target strategies in everyday clinical practice. The consequences of failing to control RA emphasizes the need to initiate appropriate treatment at the right time to prevent or delay the deterioration in health that has such a negative impact on QoL and functional ability.

Our results show that the 2 dimensions that contributed the most to a patient’s impaired QoL were pain/discomfort and depression/anxiety. Results from the EQ-5D questionnaire indicated that mobility and usual activities were also partially impaired in approximately half of the patients. Excluding mobility, none of the EQ-5D dimensions we analyzed showed notable improvement throughout the observational period, even under different treatment regimens. The absence of improvement may be due to the study design in that these patients were mostly experiencing moderate-to-severe disease and were on stable treatments throughout follow-up. Indeed, data from the Hellenic Registry of Biologic Therapies have shown that an improvement in EQ-5D mainly occurs in patients starting biologic therapies [23]. In general, our observations suggest that current RA management should focus on timely and effective treatment in order to address the full range of disease symptoms. Given the positive relationship between QoL and functional ability, an improvement in the latter should lead to an improvement in the former.

When looking at patients’ perspectives of their functional ability, 31–33% had total HAQ scores >1.0 during the study period, which represents a level of physical dysfunction that patients do not tolerate well [24, 25]. Patients who were treated with bDMARDs had higher HAQ scores than those treated with non-bDMARDs, possibly because physicians prescribed more aggressive therapies to the patients with more severe and established RA. The HAQ scores reported in our study are comparable to those previously reported in Greek patients under biologic therapies and followed by the Hellenic Registry of Biologic Therapies [26].

Inadequately controlled RA has long-term consequences that should not be overlooked, and which span from restriction of mobility, pain, and depression to premature retirement. Premature retirement as a direct consequence of having RA occurred in 38.5% of the patients in the present study. The proportion of unemployed patients in this study was in line with that reported in the literature, in which a 40% drop in employment has been observed within 5 years of being diagnosed with RA [27–29]. Even within the employed population, periods of absence and shortened working days were observed. At work, the most common problem associated with having RA was an inability to work at a normal tempo or the need to postpone tasks. At home, 60% of patients found it hard to perform normal housework and other duties to such an extent that, in most cases, they required assistance from family members or paid staff. The incidence of hospitalization due to exacerbation of RA during the study period was a further indicator of unmet needs in patients with established RA.

These effects upon a patients ability to perform everyday tasks and employed work, when taken together with the higher rate of comorbidities in patients with RA compared with the general population, have a further negative impact on QoL [30] and place an additional burden on patients, their families, and the healthcare system. It is well known that increases in healthcare resource utilization are associated with the duration and severity of RA, and impaired QoL aggravates the economic burden associated with management of the disease [31]. This suggests that intervention to achieve disease control as early as possible currently represents the best strategy to prevent deterioration of functional ability.

The present study has some limitations. Although the study was designed to include patients in all clinical stages of RA, the majority had long-standing disease and may not be representative of the overall population of patients with RA in Greece, particularly those patients in the early stages and with milder symptoms. This bias is inherent to the study because patients were recruited from referral centers where patients with more severe phenotypes are followed. In addition, patient-reported outcomes based on questionnaires warrant caution because they contain a certain degree of recall bias. The possibility of reviewer bias in the interpretation of questionnaires was minimized by the use of standardized questionnaires validated in the Greek population. An additional limitation is that the resource utilization observed in the present study cannot be considered exclusively related to RA because of the high prevalence of comorbidities in this patient population. The presence of patient comorbidities at baseline was not included as a covariate in the comparisons of EQ-5D or HAQ and therefore it cannot be ruled out that these may have contributed to the results presented, although it is probable that any such comorbidities would have been due to the presence of long-term RA rather than independent from it. Finally, the study described in the present analysis was conducted from 2010 to 2012 and therefore the data presented may not entirely reflect the contemporary clinical situation in this setting.

## Conclusion

This observational study of patients with long-standing RA in Greece who attended tertiary referral centers reveals that unmet needs may exist in this population. Failure to optimize disease control in patients with long-standing RA results in substantial adverse consequences on patient outcomes and places a considerable burden on patients, their families, and the healthcare system. Effective RA treatment aimed towards a predefined target, and which is applied in a timely manner during the course of the disease, is recommended in order to improve and sustain clinical and patient-reported outcomes as well as to control disease-related resource utilization.

## Additional file

Additional file 1: **Table S1.** Final fitted mixed-effects model across study time points for EQ-5D index score. **Table S2.** HAQ results in the overall cohort at each study visit. **Table S3.** HAQ results over time according to use of bDMARDs (including categoric data). **Table S4.** Final fitted mixed-effects model across study time points for HAQ-DI score. (DOCX 50 kb)

## Abbreviations

bDMARD: Biologic disease-modifying antirheumatic drug
csDMARD: Conventional synthetic disease-modifying antirheumatic drug
EQ-5D: Euro Quality of Life-5 dimensions
HAQ: Health Assessment Questionnaire
HAQ-DI: Health Assessment Questionnaire Disability Index
NSAID: Nonsteroidal anti-inflammatory drug
QoL: Quality of life
RA: Rheumatoid arthritis
SD: Standard deviation
tsDMARD: Targeted synthetic disease-modifying antirheumatic drug

## References

[1] McInnes IB, Schett G (2011). The pathogenesis of rheumatoid arthritis. N Engl J Med.

[2] Hurst NP, Kind P, Ruta D, Hunter M, Stubbings A (1997). Measuring health-related quality of life in rheumatoid arthritis: validity, responsiveness and reliability of EuroQol (EQ-5D). Br J Rheumatol.

[3] Kosinski M, Kujawski SC, Martin R, Wanke LA, Buatti MC, Ware JE, Perfetto EM (2002). Health-related quality of life in early rheumatoid arthritis: impact of disease and treatment response. Am J Manag Care.

[4] Matcham F, Scott IC, Rayner L, Hotopf M, Kingsley GH, Norton S, Scott DL, Steer S (2014). The impact of rheumatoid arthritis on quality-of-life assessed using the SF-36: a systematic review and meta-analysis. Semin Arthritis Rheum.

[5] Furuya H, Kasama T, Isozaki T, Umemura M, Otsuka K, Isojima S, Tsukamoto H, Tokunaga T, Yanai R, Takahashi R (2013). Effect of TNF antagonists on the productivity of daily work of patients with rheumatoid arthritis. J Multidiscip Healthc.

[6] Liao KP, Solomon DH (2013). Traditional cardiovascular risk factors, inflammation and cardiovascular risk in rheumatoid arthritis. Rheumatology (Oxford).

[7] Humphreys J, Hyrich K, Symmons D (2016). What is the impact of biologic therapies on common co-morbidities in patients with rheumatoid arthritis?. Arthritis Res Ther.

[8] Innala L, Sjöberg C, Möller B, Ljung L, Smedby T, Södergren A, Magnusson S, Rantapää-Dahlqvist S, Wållberg-Jonsson S (2016). Co-morbidity in patients with early rheumatoid arthritis - inflammation matters. Arthritis Res Ther..

[9] Matcham F, Rayner L, Steer S, Hotopf M (2014). The prevalence of depression in rheumatoid arthritis: a systematic review and meta-analysis: reply. Rheumatology (Oxford).

[10] Cooper NJ (2000). Economic burden of rheumatoid arthritis: a systematic review. Rheumatology (Oxford).

[11] Espinoza F, Fabre S, Pers YM (2016). Remission-induction therapies for early rheumatoid arthritis: evidence to date and clinical implications. Ther Adv Musculoskelet Dis.

[12] Monti S, Montecucco C, Bugatti S, Caporali R (2015). Rheumatoid arthritis treatment: the earlier the better to prevent joint damage. RMD Open.

[13] Singh JA, Saag KG, Bridges SL, Akl EA, Bannuru RR, Sullivan MC, Vaysbrot E, McNaughton C, Osani M, Shmerling RH (2016). 2015 American College of Rheumatology Guideline for the treatment of rheumatoid arthritis. Arthritis Care Res (Hoboken).

[14] Smolen JS, Landewé R, Bijlsma J, Burmester G, Chatzidionysiou K, Dougados M, Nam J, Ramiro S, Voshaar M, van Vollenhoven R (2017). EULAR recommendations for the management of rheumatoid arthritis with synthetic and biological disease-modifying antirheumatic drugs: 2016 update. Ann Rheum Dis.

[15] Schipper LG, Fransen J, den Broeder AA, Van Riel PL (2010). Time to achieve remission determines time to be in remission. Arthritis Res Ther..

[16] Klarenbeek NB, Güler-Yüksel M, van der Kooij SM, Han KH, Ronday HK, Kerstens PJ, Seys PE, Huizinga TW, Dijkmans BA, Allaart CF (2011). The impact of four dynamic, goal-steered treatment strategies on the 5-year outcomes of rheumatoid arthritis patients in the BeSt study. Ann Rheum Dis.

[17] EuroQol Group (1990). EuroQol—a new facility for the measurement of health-related quality of life. Health Policy.

[18] Kontodimopoulos N, Pappa E, Niakas D, Yfantopoulos J, Dimitrakaki C, Tountas Y (2008). Validity of the EuroQoL (EQ-5D) instrument in a Greek general population. Value Health.

[19] Chatzitheodorou D, Kabitsis C, Papadopoulos NG, Galanopoulou V (2008). Assessing disability in patients with rheumatic diseases: translation, reliability and validity testing of a Greek version of the Stanford health assessment questionnaire (HAQ). Rheumatol Int.

[20] Kobelt G, Lindgren P, Lindroth Y, Jacobson L, Eberhardt K (2005). Modelling the effect of function and disease activity on costs and quality of life in rheumatoid arthritis. Rheumatology (Oxford).

[21] Yelin E, Wanke LA (1999). An assessment of the annual and long-term direct costs of rheumatoid arthritis: the impact of poor function and functional decline. Arthritis Rheum.

[22] Mathews AL, Coleska A, Burns PB, Chung KC (2016). Evolution of patient decision-making regarding medical treatment of rheumatoid arthritis. Arthritis Care Res (Hoboken)..

[23] Boubouchairopoulou N, Flouri I, Drosos AA, Boki K, Settas L, Zisopoulos D, Skopouli FN, Papadopoulos I, Iliopoulos A, Kyriopoulos J (2016). Treatment with the first TNF inhibitor in rheumatoid arthritis patients in the Hellenic registry of biologic therapies improves quality of life especially in young patients with better baseline functional status. Clin Exp Rheumatol.

[24] Wells GA, Tugwell P, Kraag GR, Baker PR, Groh J, Redelmeier DA (1993). Minimum important difference between patients with rheumatoid arthritis: the patient's perspective. J Rheumatol.

[25] Westhovens R, Cole JC, Li T, Martin M, Maclean R, Lin P, Blaisdell B, Wallenstein GV, Aranda R, Sherrer Y (2006). Improved health-related quality of life for rheumatoid arthritis patients treated with abatacept who have inadequate response to anti-TNF therapy in a double-blind, placebo-controlled, multicentre randomized clinical trial. Rheumatology (Oxford).

[26] Flouri I, Markatseli TE, Voulgari PV, Boki KA, Papadopoulos I, Settas L, Zisopoulos D, Skopouli FN, Iliopoulos A, Bertsias GK (2014). Comparative effectiveness and survival of infliximab, adalimumab, and etanercept for rheumatoid arthritis patients in the Hellenic registry of biologics: low rates of remission and 5-year drug survival. Semin Arthritis Rheum.

[27] Scott DL, Smith C, Kingsley G (2005). What are the consequences of early rheumatoid arthritis for the individual?. Best Pract Res Clin Rheumatol.

[28] Young A, Dixey J, Cox N, Davies P, Devlin J, Emery P, Gallivan S, Gough A, James D, Prouse P (2000). How does functional disability in early rheumatoid arthritis (RA) affect patients and their lives? Results of 5 years of follow-up in 732 patients from the early RA study (ERAS). Rheumatology (Oxford).

[29] Young A, Dixey J, Kulinskaya E, Cox N, Davies P, Devlin J, Emery P, Gough A, James D, Prouse P (2002). Which patients stop working because of rheumatoid arthritis? Results of five years' follow up in 732 patients from the early RA study (ERAS). Ann Rheum Dis.

[30] Havens E, Slabaugh SL, Helmick CG, Cordier T, Zack M, Gopal V, Prewitt T (2017). Comorbid arthritis is associated with lower health-related quality of life in older adults with other chronic conditions, United States, 2013-2014. Prev Chronic Dis.

[31] Kavanaugh A (2007). Economic consequences of established rheumatoid arthritis and its treatment. Best Pract Res Clin Rheumatol.

