# Supplementary material for: Health outcomes and unmet needs in patients with long-standing rheumatoid arthritis attending tertiary care in Greece: a cohort study
Source: Health Qual Life Outcomes. 2019 Apr 29;17:73. doi: 10.1186/s12955-019-1127-8 (PMC6489275; doi:10.1186/s12955-019-1127-8)
Supplement: Supplementary file 1 — Table S1. Final fitted mixed-effects model across study time points for EQ-5D index score. Table S2. HAQ results in the overall cohort at each study visit. Table S3. HAQ results over time according to use of bDMARDs (including categoric data). Table S4. Final fitted mixed-effects model across study time points for HAQ-DI score. (DOCX 50 kb) [file 12955_2019_1127_MOESM1_ESM.docx]

**Health Outcomes and Unmet Needs in Patients With Long-Standing Rheumatoid Arthritis Attending Tertiary Care in Greece: a Cohort Study**

**Supplementary information for Boumpas et al. (2019)**

**SUPPLEMENTARY RESULTS**

**Disease-related parameters during follow-up**

*Changes in categoric HAQ data over time*

Throughout the follow-up period, there were 10.5–12.4% of patients who had a health status that was poor (total HAQ score >1.5), 18.1–22.9% who had a health status that was moderate (total HAQ score of 1.0–1.5), and 66.7–69.5% who had a mild-to-moderate health status (total HAQ score of <1.0; Additional file 1: Table S2). At 9 months, 67.2% of patients in the bDMARD subgroup and 74.4% of patients in the non-bDMARD subgroup had a mild-to-moderate health status (total HAQ score <1.0) compared with 61.8% and 73.3% at baseline, respectively (Additional file 1: Table S3).

**Table S1** Final fitted mixed-effects model across study time points for EQ-5D index score.

| Parameter | Estimate | SE | p-value* | 95% CI |
| --- | --- | --- | --- | --- |
| Non-biologic DMARDs | 0.107 | 0.021 | <0.0001 | 0.066, 0.148 |
| Gender† | −0.195 | 0.025 | <0.0001 | −0.243, −0.146 |
| Smoking status‡ | 0.033 | 0.014 | 0.021 | 0.004, 0.060 |

*The global p-values for each of the interaction terms were evaluated: Visit*Age p = 0.110, Visit*Smoking status p = 0.252, and Visit*Gender p = 0.101.

†Reference category was ‘Male’.

‡Reference category was ‘Current smoker’.

*CI* confidence interval, *DMARD* disease-modifying antirheumatic drug, *EQ-5D* Euro Quality of Life-5 dimensions, *SE* standard error

**Table S2** HAQ results in the overall cohort at each study visit.

|  | **Baseline**  **(*N* = 210)** | **3 months**  **(*N* = 210)** | **6 months**  **(*N* = 210)** | **9 months**  **(*N* = 210)** |
| --- | --- | --- | --- | --- |
| HAQ total score, mean ± SD | 0.75 ± 0.63 | 0.70 ± 0.62 | 0.70 ± 0.60 | 0.70 ± 0.61 |
| HAQ categorized, n (%) |  |  |  |  |
| *<0.5* | 79 (37.6) | 89 (42.4) | 81 (38.6) | 79 (37.6) |
| *0.5–<1.0* | 61 (29.0) | 51 (24.3) | 62 (29.5) | 67 (31.9) |
| *1.0–<1.5* | 39 (18.6) | 48 (22.9) | 45 (21.4) | 38 (18.1) |
| *1.5–<2.0* | 21 (10.0) | 12 (5.7) | 14 (6.7) | 16 (7.6) |
| *2.0–<2.5* | 7 (3.3) | 6 (2.9) | 5 (2.4) | 8 (3.8) |
| *≥2.5* | 3 (1.4) | 4 (1.9) | 3 (1.4) | 2 (1.0) |
| *HAQ* Health Assessment Questionnaire | | | | |

**Table S3** HAQ results over time according to use of bDMARDs (including categoric data).

|  | Baseline | | 3 months | | 6 months | | 9 months | | |
| --- | --- | --- | --- | --- | --- | --- | --- | --- | --- |
|  | **Non-bDMARDs**  *N* = 86 | **bDMARDs**  *N* = 123 | **Non-bDMARDs**  *N* = 68 | **bDMARDs**  *N* = 115 | **Non-bDMARDs**  *N* = 73 | **bDMARDs**  *N* = 121 | **Non-bDMARDs**  *N* = 78 | | **bDMARDs**  *N* = 119 |
| Total HAQ score,  mean ± SD | 0.68 ± 0.64 | 0.81 ± 0.63 | 0.57 ± 0.62 | 0.75 ± 0.59 | 0.53 ± 0.54 | 0.77 ± 0.62 | 0.54 ± 0.53 | 0.78 ± 0.62 | |
| Adjusted total HAQ score*,  Least-squares mean ± SE | 0.67 ± 0.08 | 0.80 ± 0.07 | 0.58 ± 0.07 | 0.75 ± 0.06 | 0.52 ± 0.07 | 0.77 ± 0.05 | 0.58 ± 0.07 | 0.78 ± 0.05 | |
| HAQ categorized, *n* (%)  <0.5  0.5–<1.0  1.0–<1.5  1.5–<2.0  2.0–<2.5  ≥2.5 | 37 (43.0)  26 (30.2)  13 (15.1)  6 (7.0)  2 (2.3)  2 (2.3) | 41 (33.3)  35 (28.5)  26 (21.1)  15 (12.2)  5 (4.1)  1 (0.8) | 37 (54.4)  12 (17.6)  14 (20.6)  2 (2.9)  2 (2.9)  1 (1.5) | 41 (35.7)  32 (27.8)  29 (25.2)  8 (7.0)  3 (2.6)  2 (1.7) | 37 (50.7)  18 (24.7)  14 (19.2)  2 (2.7)  2 (2.7)  0 (0.0) | 39 (32.2)  39 (32.2)  28 (23.1)  10 (8.3)  3 (2.5)  2 (1.7) | 38 (48.7)  20 (25.6)  15 (19.2)  4 (5.1)  1 (1.3)  0 (0.0) | 36 (30.3)  44 (37.0)  21 (17.6)  11 (9.2)  6 (5.0)  1 (0.8) | |

*HAQ scores adjusted for baseline covariates: age, gender, BMI, and the interaction term of Visit*BMI.

*bDMARD* biologic disease-modifying antirheumatic drug, *BMI* body mass index, *HAQ* Health Assessment Questionnaire, *SD* standard deviation, *SE* standard error

**Table S4** Final fitted mixed-effects model across study time points for HAQ-DI score.

| Parameter | Estimate | SE | p-value* | 95% CI |
| --- | --- | --- | --- | --- |
| Non-biologic DMARDs | −0.186 | 0.044 | <0.0001 | −0.272, −0.099 |
| Age | 0.006 | 0.002 | 0.001 | 0.002, 0.009 |
| Gender† | 0.359 | 0.050 | <0.0001 | 0.261, 0.458 |
| BMI | −0.009 | 0.004 | 0.012 | −0.016, −0.002 |
| Visit=Baseline*BMI‡ | 0.039 | 0.010 | <0.0001 | 0.019, 0.059 |
| Visit=3 months*BMI‡ | 0.007 | 0.005 | 0.201 | −0.004, 0.017 |
| Visit=6 months*BMI‡ | 0.004 | 0.005 | 0.454 | −0.006, 0.014 |

*The global p-values for each of the interaction terms were evaluated: Visit*Age p = 0.310, Visit*BMI p = 0.001, and Visit*Gender p = 0.808. Only significant interaction terms were included in the final model.

†Reference category was ‘Male’.

‡Reference category was the Month 9 visit.

*BMI* body mass index, *CI* confidence interval, *DMARD* disease-modifying antirheumatic drug, *HAQ-DI* Health Assessment Questionnaire-Disability Index, *SE* standard error
